# Antithyroid drugs

**DOI:** 10.1186/1756-6614-8-S1-A12

**Published:** 2015-06-22

**Authors:** Helena Jastrzębska

**Affiliations:** 1 Endocrinology Department CMKP; ul. Marymoncka 99, 01–813 Warsaw, Poland

## 

Antithyroid drugs were introduced into medical practice in 1945. These are tionamide derivatives that contain a sulfhydryl group - preparations include methimazole (MMI) and carbimazole (CMI) or thiourea derivatives - preparation propylthiouracyl (PTU). CMI is an inactive form that is converted in the body to MMI.

Both MMI and PTU are available in Europe and Asia and in some countries CMI is available too. In the USA, only MMI and PTU are available. Administered orally, they reach maximum serum concentration after 1-2 hours. MMI does not bind to serum proteins and remains in the free form, while 80-90% of PTU binds to serum proteins. These drugs actively accumulate in the thyroid gland against the gradient concentration. The metabolic effect of PTU lasts 12-24 h, therefore this drug has to be administered 2-3 times per day. MMI maintains biological activity for more than 24 hours, and in this context may be administered once a day. Both preparations inhibit iodination of tyrosyl residues in thyroglobulin driven by thyroid peroxidase, thereby inhibiting the synthesis of thyroid hormones. They only block formation of new thyroid hormones but do not remove thyroid hormones which are already in the thyroid or in the blood stream. Antithyroid drugs also have an immunosuppressive effect, which is manifested by reduction of TSH receptor antibody (TRAb) serum level, induction of intrathyroidal lymphocytes apoptosis, increasing of the number of suppressor T cells and reduction of the number of helper T cells as well. This facilitates achievement of remission of Graves' disease. PTU may also act in the peripheral tissues by inhibiting conversion of thyroxine to triiodothyronine.

Antithyroid drugs are recommended for treatment of hyperthyroidism caused by overproduction of hormones in children, adults and pregnant women. They can be used as a long-term essential treatment for Graves’ disease and as a short-term treatment to prepare the patients with Graves’ disease or toxic nodular goiter for thyroid surgery or radioiodine.

In adults, the treatment of Graves’ disease usually lasts 12-18 months but it is also possible to order longer administration of low doses of antithyroid drugs. The initial dose of the antithyroid drugs depends on the severity of hyperthyroidism, the size of the thyroid gland and the supply of iodine. Starting dose of MMI is usually 15-30 mg, administered in a single dose, or equivalent by 150-300mg PTU in three divided doses. After 4-12 weeks euthyroidism is usually achieved and antithyroid drug dose can be reduced to 5-10mg MMI or 100-200mg PTU. Further treatment with low doses is continued for 1.5 years or longer. Long lasting remission is achieved in approximately 30% of patients. Relapse of hyperthyroidism occurs usually in the first 6 months after cessation of treatment. Then the recurrence rate decreases, and reaches 50-60% of patients after 1-2 years following cessation of treatment. A particular high risk of relapse concerns patients who remain TRAb positive at the end of therapy.

The purpose of preparation for radioiodine therapy or thyroid surgery is to bring to the euthyroid state, confirmed by normal free triiodotyronine (fT3) and free thyroxine (fT4) serum levels, which usually takes a few weeks or months.

The initial dose of antithyroid drugs in children depends on the body weight and do not exceed 0.5-1 mg/kg MMI or 5-10 mg/kg/kg PTU. Antithyroid treatment in children should be continued for many years, at least for 24 months.

Antithyroid drug therapy is associated with the risk of side effects. Minor side effects involving 15% patients are itching, rash, urticaria, joint pain, swelling, abnormal sense of taste or smell, nausea, or vomiting. These symptoms are not life-threatening and do not require discontinuation of antithyroid drug. Switching to another drug, dose reduction or antihistamine addition may be helpful. Major side effects are potentially life-threatening or even lethal and occur in less than 1% of patients. These include agranulocytosis, which can occur during therapy with MMI in 0.35% of patients, and with PTU in 0.37%. Occasionally, aplastic anemia and vasculitis or hepatitis develop after PTU, particularly in children. For this reason the MMI is the first choice drug for treating hyperthyroidism. In cases of serious side effects antithyroid drugs must be immediately discontinued and such patient should be hospitalized. Case report side effects are pancreatitis and hypoglycemia after MMI. This latter condition is connected with the appearance of anti-insulin antibodies.

Both drugs may have teratogenic effects. During the first trimester of pregnancy PTU is preferred because of lower risk of fetus defects. Congenital malformations associated with the use of MMI during pregnancy are called methimazole embryopathy, which include aplasia cutis congenita, choanal atresia and intestinal anomalies. Antithyroid drugs are secreted in breast milk in low concentrations and therefore the therapy is not contraindicated in breastfeeding women.

Antithyroid drugs are used to treat the thyroid storm. In such cases high doses of MMI reaching 120 mg/d or PTU reaching 1200mg/d are recommended.
